# The Role of Th17 in Neuroimmune Disorders: Target for CAM Therapy. Part II

**DOI:** 10.1093/ecam/nep063

**Published:** 2011-06-16

**Authors:** Aristo Vojdani, Jama Lambert

**Affiliations:** Immunosciences Lab., Inc., Los Angeles, CA 90035, USA

## Abstract

Decades of research went into understanding the role that Th1 autoreactive T-cells play in neuroinflammation. Here we describe another effector population, the IL-17-producing T-helper lineage (Th17), which drives the inflammatory process. Through the recruitment of inflammatory infiltration neutrophils and the activation of matrix metalloproteinases, IL-17, a cytokine secreted by Th17 cells, contributes to blood-brain barrier breakdown and the subsequent attraction of macrophages and monocytes into the nervous system. The entry of cells along with the local production of inflammatory cytokines leads to myelin and axonal damage. This activation of the inflammatory response system is induced by different pathogenic factors, such as gut bacterial endotoxins resulting in progressive neurodegeneration by Th17 cells. Through the understanding of the role of bacterial endotoxins and other pathogenic factors in the induction of autoimmune diseases by Th17 cells, CAM practitioners will be able to design CAM therapies targeting IL-17 activity. Targeted therapy can restore the integrity of the intestinal and blood-brain barriers using probiotics, *N*-acetyl-cysteine, **α**-lipoic acid, resveratrol and others for their patients with autoimmunities, in particular those with neuroinflammation and neurodegeneration.

## 1. Introduction

In the previous article, we established the differentiation of activated T-helper cells into T-helper-17 (Th17) and the pathogenic role of interleukin-17 (IL-17) in neuroimmune disorders. Here we review the inflammatory pathophysiology of intestinal barrier permeability, which can lead to the breach of the blood-brain barrier (BBB). The tight junctions of the intestinal barrier may open due to environmental stressors, which can upset the microflora homeostasis thereby producing endotoxins that activate proinflammatory cytokine IL-1*β*. The upregulation of IL-1*β* begins a cascade of the inflammatory response system, resulting in high levels of IL-17-producing cells in various tissues. Additionally, the stressors that are capable of opening the epithelial tight junction barrier are also able to breakdown the BBB. Once inside the central nervous system, Th17 deposits IL-17 leading to CNS inflammation, which attracts additional inflammatory substances [1–6]. Neuropeptide substance P (SP), along with its high affinity receptor neurokinin-1 (NK-1), activates NF-*κ*B, a facilitator in the production of proinflammatory cytokines, and SP also reduces the production of immunoregulatory IL-10 and TGF-*β* [7–9]. Therefore, daily environmental stressors can disrupt gut function, which can lead to breakdown of the BBB and subsequent induction of neuro-inflammation. Our objective is to provide CAM practitioners with additional targets in the assessment and treatment of patients presenting with autoimmune, inflammatory or neuroimmune disorders. Therapeutic protocols may include integrative use of pharmaceuticals in conjunction with CAM therapies entailing body, mind and spirit, for the repair of gut barrier dysfunction first, systemic inflammation second and lastly, enhanced BBB permeability.

## 2. Intestinal Epithelial Tight Junction Permeability and Disruption of BBBs by Th17 Cells

Environmental factors such as stress, drugs and xenobiotics can induce imbalanced gut floral and bacterial translocation. These stressors mediate intestinal permeability with subsequent development of intestinal inflammation [1, 2]. Bacterial antigens, in particular lypopolysaccharide (LPS) prepared from enteropathogenic *Escherichia coli, Clostridium difficile, Klebsiella pneumoniae, Pseudomonas aeruginosa, Hafnia alvei, Citrobacter koseri* and others, can induce an increase in intestinal tight junction (TJ) permeability [3–5]. Imbalanced gut flora can result in the production of LPS by a variety of gram-negative bacteria. The release of LPS induces mucosal immune dysregulation and the production of proinflammatory cytokines, in particular IL-1*β*, IL-6 and TNF-*α* [6].

IL-1*β* production induced by LPS can promote inflammatory response in various tissues [6, 10–12]. This enhanced production of IL-1*β* causes direct disruption of the intestinal TJ barrier leading to paracellular permeation of luminal antigens into circulation, which may contribute to systemic inflammation [13–17]. Until recently, the intracellular mechanism that mediates IL-1*β* modulation of intestinal TJ barrier was not clear [18]; therefore, several studies tried to elucidate this mechanism of action [19–21].

**Figure 1:**
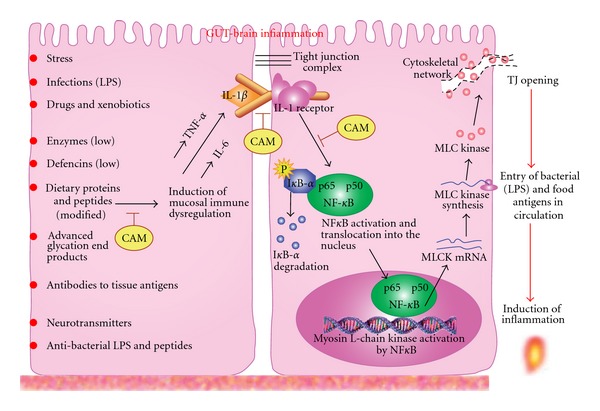
Proposed scheme of the induction by environmental factors of mucosal immune dysregulation and the production of inflammatory cytokines, resulting in the epithelial cell tight junction opening and the entry of bacterial and food antigens in the circulation and further activation of inflammatory cascade in the blood and in different tissues. CAM targets may include one or a combination of the following: inhibition of proinflammatory cytokines, inhibition of IL-1*β* binding to its receptor and inhibition of NF-*κ*B activation.

Previously it was shown that IL-1*β* causes an increase in NF-*κ*B activation, which was required for IL-1*β*-induced increase in TJ permeability [13]. In their recent article [18], Al-Sadi and colleagues indicate that IL-1*β* causes a rapid activation of NF-*κ*B and inhibition of NF-*κ*B activation prevented IL-1*β*-induced MLCK protein expression and TJ permeability. These findings indicate that NF-*κ*B activation of TJ barrier is mediated in part by an increase in MLCK protein and mRNA expression [18]. To further identify the role of NF-*κ*B in mediating IL-1*β*-induced MLCK expression and TJ permeability, researchers used NF-*κ*B P65 silencing studies using SiRNA. The SiRNA silencing of NF-*κ*B P65 expression completely inhibited the IL-1*β*-induced protein expression and its drop in transepithelial electrical resistance of intestinal monolayers, which confirmed the regulatory role of NF-*κ*B in the mediation of IL-1*β*-induced MLCK mRNA transcription and protein expressions [22]. IL-1*β* binding to IL-1*β* receptor on epithelial cells causes a rapid activation of NF-*κ*B, I*κ*B-*α* phosphorylation and I*κ*B-*α* degradation. NF-*κ*B activation leads to its translocation into the nucleus, resulting in stimulation of myosin L-chain kinase activation, increase in myosin L-chain kinase (MLCK) RNA and MLC kinase synthesis [22–26]. This increase in MLCK activity leads to the opening of TJ barriers and the entry of bacterial endotoxins (LPS), and dietary proteins and peptides into the submucosa, regional lymph nodes and blood stream, followed by the initiation of inflammation in circulation and in different tissues (Figure 1).

The understanding of cellular mechanisms, which lead to intestinal TJ barrier defect during inflammatory conditions, can assist in designing potential CAM therapeutic interventions targeting IL-1*β* NF-*κ*B or other molecules necessary for repairing the TJ barriers during the inflammatory state.

## 3. Support for the LPS Mechanism of Action in the Induction of the Inflammatory Response System (IRS)

Recently, it was shown that intestinal barrier dysfunction, with an increased translocation of LPS, correlated with serum IgA and IgM against LPS and the inflammatory pathophysiology of depression and chronic fatigue syndrome [27, 28]. Normalization of increased LPS translocation is accompanied by a remission in clinical symptomatologies. Therefore, the regulation of intestinal barrier function via CAM therapies such as yoga, herbal cleanses and use of probiotics, is proven to be an important first step in the recovery of patient health.

## 4. The Effect of LPS and Inflammatory Cytokines on the Induction of BBB Disruption and Neuroinflammation

The BBB separates blood leukocytes, which normally respond to necrotic injury, from the brain parenchyma where necrotic cell death might take place in response to environmental factors such as infections, toxins, excitotoxicity or trauma [24]. The BBB is composed of two layers. The first layer consists of microvascular endothelial cells, which have abundant tight junctions with structural similarity to that of intestinal epithelial cells [27]. The second layer is the glia limitans, which is formed by glial foot processes [26]. The perivascular space between the endothelial cells and astrocytes is populated by macrophages, which behave like immature dendritic cells [26]. Therefore, factors capable of opening the epithelial TJ barrier are able to destroy both the BBB and neural tissue [27–30]. This includes bacterial endotoxins, proinflammatory cytokines, enzyme and effector cells Th1 and Th17, which are essential to the central nervous system inflammation [27–31].

It is firmly established that disruption of the BBB by endotoxins, cytokines, chemokines, adhesion molecules and others, and the trafficking of autoreactive T-cells from the systemic compartment into the central nervous system play an important role in the development of MS lesions [32–34]. However, when a comparison was made between human Th1 versus Th17 lymphocytes, human Th17 lymphocyte migrated faster across the BBB than Th1 lymphocytes. Indeed a significant number of IL-17- and IL-22-expressing CD4^+^CD45RO^+^ memory lymphocytes upon their migration across BBB expressed IL-17^+^ and IL-22^+^ markers, which confirmed the ability of Th17 lymphocytes to cross the BBB *in vitro* and *in vivo* [31]. The BBB endothelial cells expressed IL-17R and IL-22R, which are used by Th17 lymphocytes to infiltrate the BBB endothelial cells (ECS). This diffusion of cells or antigens, such as bovine serum albumin (BSA), across the BBB was enhanced significantly when IL-17 and IL-22 were added to monolayers of human BBB-ECS. This enhanced permeability of BBB-ECS correlated with a decrease in the expression of occludin and zonulin, the two important tight junction proteins [35].

To validate observations of Th17 involvement under human neuroinflammatory conditions, brain sections from humans with MS and from unaffected controls were immunostained for IL-17, IL-22 and CD45RO. Many CD45RO^+^ cells immunopositive for IL-17 or IL-22 were shown in patients with MS but not in controls [31]. These results emphasized the importance of Th17 infiltration into the CNS for the development of brain lesions in MS. In a search for a mechanism of lesion induction by Th17 cells in MS, it was shown that in addition to IL-17 production and its proinflammatory action, up to 60% of cells expressing IL-17 and also IL-22 expressed the cytolytic enzyme called granzyme B. Furthermore, when granzyme B-Th17 cells were added to neuron-enriched cultures, a significant number of neurons underwent programed cell death [31–34]. Therefore, Th17 cell production of mediators such as granzyme B contributes to their highly encephalitogenecity.

**Figure 2:**
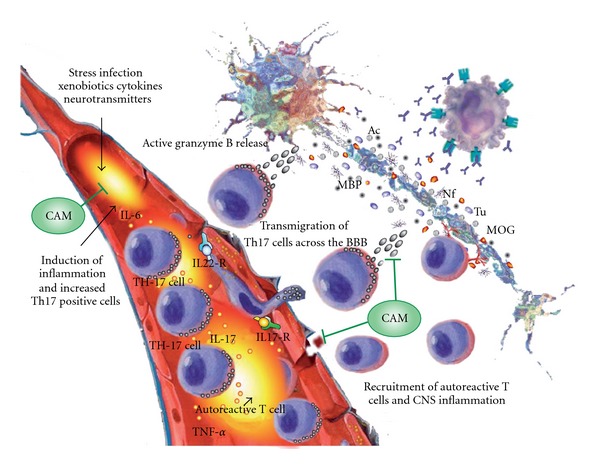
The role of Th17 lymphocytes in the pathogenesis of inflammatory and neuroimmunological disorders. Environmental factors' induction of inflammatory response, production of cytokines and increase in the number of Th17 positive cells in circulation. Expression of IL-17 and IL-22 receptors on blood-brain barrier endothelial cells result in the binding of Th17 cells to BBB tight junctions. This disrupts the tight junctions, and the Th17 cells then transmigrate across the BBB, setting the stage for the killing of neurons by the release of granzyme B. CAM protocols may be used to block the inflammatory cascade induced by infection. Additionally, CAM therapies resulting in the repair of the BBB and the inhibition of lymphocyte transmigration can reduce neuro-inflammation.

Since microglia plays a significant role in MS, to further strengthen the role of IL-17 in CNS inflammation, microglia were treated with IL-17. Treatment with IL-17 upregulated the microglial production of IL-6, MIP-2, nitric oxide, adhesion molecules and neurotrophic factors [35–39]. It was also found that IL-17 could be produced by microglia in response to IL-1*β* or IL-23. Because microglia produces IL-1*β* and IL-23, these cytokines may act in an autocrine manner to express IL-17 on microglia, and thereby contribute to autoimmune disease in the CNS [39]. These results strongly suggest that the cells co-expressing IL-17, IL-22 and granzyme B through the action of IL-17 and IL-22 play a significant role in the induction and breach in the BBB and the permeabilization of BBB to circulating CD4^+^ lymphocytes and soluble molecules resulting in CNS inflammation [37]. The role of Th17 lymphocytes in the pathogenesis of inflammatory and neuroimmunological disorders is shown in Figure 2. This role of Th17 cells and the IL-17 produced by them in neuroinflammation make these novel CD4 cells a suitable target for CAM treatment of neuroimmune disorders that affect a significant percentage of the population. Steps to tackling neuroinflammation begin with the repair of gastrointestinal barrier dysfunction, followed by dampening systemic inflammation and ending with the restoration of the BBB.

## 5. Direct Effect of Bacterial Antigens on the Production of IL-17 and Other Cytokines by Microglia and Astrocytes

It has become increasingly apparent that resident glial cells such as microglia and astrocytes, play an important role in the initiation and progression of immune responses following pathogen invasion or their antigens [40–42]. Such infections are associated with high levels of inflammatory cytokines including IL-1*β*, IL-6, TNF-*α* and IL-17 in the CNS that result in neurological dysfunction. Microglia and astrocytes can perceive bacteria through microbial pattern recognition receptors, such as TLR and nucleotide-binding domain leucine-rich repeat containing receptor families. Binding of bacteria to these receptors promotes recruitment and antigen-specific activation of infiltrating leukocytes [42, 43].

A compelling body of evidence has accumulated in the literature to show that substance P (SP) plays an important role in the augmentation of inflammatory immune responses in the gastrointestinal tract, the skin and other peripheral sites [7, 8]. Neuropeptide SP can also modulate the function of myeloid cells, such as dendritic cells and macrophages via a high affinity receptor called neurokinin-1 (NK-1); interestingly, both bacterial antigens LPS and SP with NK-1R activate the transcription factor NF-*κ*B, which facilitates the production of key proinflammatory cytokines such as TNF-*α*, IL-6 and IL-17. Furthermore, SP can reduce the production of immunoregulatory cytokines (TGF-*β*, IL-10) by macrophages, thereby further exacerbating inflammation [7, 8].

**Figure 3:**
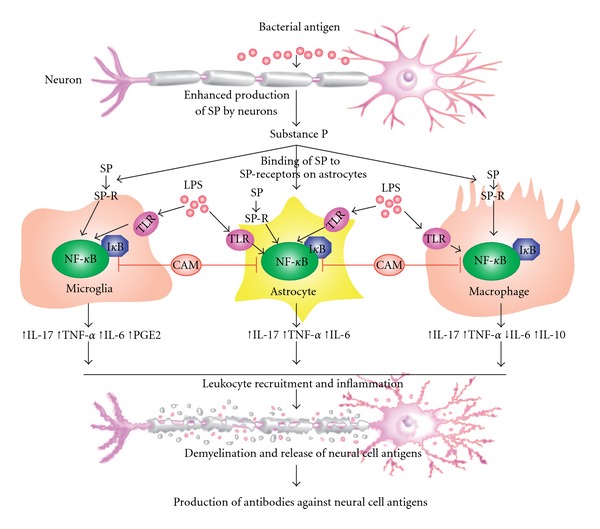
Direct effect of bacterial antigens (LPS) on the production of substance P and its exacerbation of the early inflammatory response to neural cell antigens. CAM inhibition of NF-*κ*B activation by microglia, astrocytes and macrophages may result in reduced neural cell damage.

The most abundant tachykinin in the brain, SP, with its high affinity receptor NK-1R is expressed on microglia, which shares a common myeloid lineage with macrophages and dendritic cells [9]. Furthermore, it was shown that SP can synergistically augment *Borrelia burgdorferi*-induced expression of cyclooxygenase-2 in microglia and increased secretion of inflammatory prostanoid PGE2 [44]. Recently Chauhan and colleagues [45] demonstrated that SP, in addition to PGE2, augments production of inflammatory cytokines by microglia and astrocytes following exposure to clinically relevant bacterial CNS pathogens such as *Neisseria meningitides* and *B. burgdorferi*. Taken together, these studies indicate a potentially important role for neuropeptide SP in the exacerbation of the resident glial immune response during inflammatory CNS disorders induced by infections, such as *B*. *burgdorferi* [46]. The mechanism by which bacterial antigens, through the production of SP by neurons and their binding to SP receptors, result in cytokine production and CNS inflammation is shown in Figure 3. Furthermore, the ability of CAM treatment with products, such as SP receptor antagonist for the inhibition of NF-*κ*B, or minocycline [47, 48], *α*-Lipoic acid [49], resveratrol [50] or quercetin [51] which are shown to be effective in the repair of BBB damaged by infections, may represent important new therapeutic strategies to combat potentially disabling consequences of inflammation within the brain.

Such an approach was described earlier in *eCAM Journal* under the title of “Novel diagnosis of Lyme disease: potential for CAM intervention.” This article described the mechanism by which *B*. *burgdorferi* induces inflammation by enhancing proinflammatory cytokine production, activation of matrix metalloproeinases resulting in BBB damage and chronic neuroborreliosis [46]. The article concluded that “practitioners of complementary and alternative medicine (CAM) by utilization of this analytical method will increase the accuracy of the diagnostic process and abridge the time for treatment with antibiotics, herbal medicines and nutritional supplements, resulting in improved quality of care and disease prognosis.” This approach using CAM treatment for many neuroimmune and autoimmune diseases of the CNS that start in the GI tract and manifest themselves in various tissues is further discussed in this and Part III of this article.

## 6. Conclusion

Although the assessment of Th1 and Th2 cytokines is used as a guide for intervention by some CAM practitioners in implementing therapeutic interventions, it is the T-helper-17 producing IL-17 which has emerged as the most pathogenic helper cell meriting possible CAM intervention. Beginning with IL-1*β*-induced inflammation of the gut, TJ permeability is brought on by the activation of MLCK phosphorylation of myosin L-chain, which in turn causes the contraction of peri-junctional actin-myosin filaments and the opening of intestinal barriers. Bacterial endotoxins, proinflammatory cytokines, enzymes and effector cells Th1 and Th17 are also capable of disrupting the BBB, inducing inflammation in the central nervous system. Inside the CNS, neuropeptide SP, along with NK-1R, is expressed on microglia and can modulate the function of myeloid cells. Both SP with NK-1R, and LPS activate NF-*κ*B, which facilitates the production of proinflammatory cytokines, notably IL-6, IL-17 and TNF-*α*. In addition to exacerbating inflammation, SP can also reduce the production of regulatory cytokines such as IL-10 and TGF-*β*. Effective treatment of inflammatory disorders in the gut and the nervous system requires first an understanding of the affect of cytokines on the cells and tissues around them, and second the maintenance of the delicate balance between pro-, anti- and regulatory cytokines. The measurement of multiple cytokines will present the CAM practitioner with a broader picture of the patient's inflammatory condition, and thereby would be useful in fitting a therapeutic intervention on an individual basis. As seen above, for cases of neuroimmune disorders, the modulation of IL-17, IL-6, IL-1*β*, SP and NF-*κ*B, the therapeutic support of the intestinal barrier and BBB repair are becoming important targets for CAM practitioners. Details on an array of anti-inflammatory medications, ranging from pharmaceuticals to vitamins and from botanicals to innate molecules, are discussed in Part III of this article.
